# Occurrence of *Leishmania infantum* in the central nervous system of naturally infected dogs: Parasite load, viability, co-infections and histological alterations

**DOI:** 10.1371/journal.pone.0175588

**Published:** 2017-04-18

**Authors:** Valéria da Costa Oliveira, Viviane Cardoso Boechat, Artur Augusto Velho Mendes Junior, Maria de Fátima Madeira, Luiz Claudio Ferreira, Fabiano Borges Figueiredo, Monique Paiva Campos, Francisco das Chagas de Carvalho Rodrigues, Raquel de Vasconcellos Carvalhaes de Oliveira, Maria Regina Reis Amendoeira, Rodrigo Caldas Menezes

**Affiliations:** 1 Laboratório de Pesquisa Clínica em Dermatozoonoses em Animais Domésticos, Instituto Nacional de Infectologia Evandro Chagas, Fundação Oswaldo Cruz, Rio de Janeiro, Brazil; 2 Laboratório de Vigilância em Leishmanioses, Instituto Nacional de Infectologia Evandro Chagas, Fundação Oswaldo Cruz, Rio de Janeiro, Brazil; 3 Serviço de Anatomia Patológica, Instituto Nacional de Infectologia Evandro Chagas, Fundação Oswaldo Cruz, Rio de Janeiro, Brazil; 4 Laboratório de Biologia Celular, Instituto Carlos Chagas, Fundação Oswaldo Cruz, Paraná, Brazil; 5 Laboratório de Epidemiologia Clínica, Instituto Nacional de Infectologia Evandro Chagas, Fundação Oswaldo Cruz, Rio de Janeiro, Brazil; 6 Laboratório de Toxoplasmose, Instituto Oswaldo Cruz, Fundação Oswaldo Cruz, Rio de Janeiro, Brazil; INRS - Institut Armand Frappier, CANADA

## Abstract

Zoonotic visceral leishmaniasis is caused by the protozoan *Leishmania infantum* and little is known about the occurrence and pathogenesis of this parasite in the CNS. The aims of this study were to evaluate the occurrence, viability and load of *L*. *infantum* in the CNS, and to identify the neurological histological alterations associated with this protozoan and its co-infections in naturally infected dogs. Forty-eight *Leishmania*-seropositive dogs from which *L*. *infantum* was isolated after necropsy were examined. Cerebrospinal fluid (CSF) samples were analyzed by parasitological culture, quantitative real-time PCR (qPCR) and the rapid immunochromatographic Dual Path Platform test. Brain, spinal cord and spleen samples were submitted to parasitological culture, qPCR, and histological techniques. Additionally, anti-*Toxoplasma gondii* and anti-*Ehrlichia canis* antibodies in serum and distemper virus antigens in CSF were investigated. None of the dogs showed neurological signs. All dogs tested positive for *L*. *infantum* in the CNS. Viable forms of *L*. *infantum* were isolated from CSF, brain and spinal cord in 25% of the dogs. Anti-*L*. *infantum* antibodies were detected in CSF in 61% of 36 dogs. Inflammatory histological alterations were observed in the CNS of 31% of the animals; of these, 66% were seropositive for *E*. *canis* and/or *T*. *gondii*. Amastigote forms were associated with granulomatous non-suppurative encephalomyelitis in a dog without evidence of co-infections. The highest frequency of *L*. *infantum* DNA was observed in the brain (98%), followed by the spinal cord (96%), spleen (95%), and CSF (50%). The highest *L*. *infantum* load in CNS was found in the spinal cord. These results demonstrate that *L*. *infantum* can cross the blood-brain barrier, spread through CSF, and cause active infection in the entire CNS of dogs. Additionally, *L*. *infantum* can cause inflammation in the CNS that can lead to neurological signs with progression of the disease.

## Introduction

Zoonotic visceral leishmaniasis (ZVL) is a disease of public health importance, which occurs in different countries in Latin America, Africa, Asia, and Europe [1]. The number of confirmed human cases of ZVL in Brazil was 81,722 between 1990 and 2015, an average of 3,143 new cases per year [2]. In Brazil, ZVL is caused by the protozoan *Leishmania infantum* and the sandfly *Lutzomyia longipalpis* is the main biological vector involved in the transmission of this parasite [3]. In urban areas, the dog (*Canis familiaris*) is the main reservoir of *L*. *infantum* and the source of infection of the vector [4].

Canine ZVL is characterized by a broad and variable spectrum of clinical signs [5], including neurological signs such as seizures, motor deficiencies, visual alterations, signs of cranial nerve paralysis, circling, intention tremor, dysmetria, vocalization, hemiparesis, paraparesis, paraplegia, and tetraplegia [6–13]. Additionally, histological alterations in the central nervous system (CNS) such as meningitis, choroid plexitis, neuronal degeneration, perivascular cuffs, necrosis and myelitis have been reported in infected dogs with and without neurological signs [7, 9–12, 14–19]. Analysis of the CNS of dogs infected with *L*. *infantum* revealed the presence of anti-*Leishmania* antibodies in cerebrospinal fluid (CSF) [6, 8, 14, 16, 18, 20] and of *L*. *infantum* DNA in the brain [13, 20–22] and CSF [11]. However, only few surveys exist on the occurrence and load of *L*. *infantum* in the CNS of dogs in which viable forms of the parasite are detected, especially in CSF and spinal cord.

In visceral leishmaniasis, the intact blood-brain barrier would prevent the entry of intracellular amastigotes and subsequent inflammatory cells [23]. Therefore, the detection of viable forms of the parasite in the CNS would not only confirm disruption of the blood-brain barrier, but also that the parasite can cause active infection, being able to multiply and to induce lesions. Furthermore, it is still unclear whether the inflammation observed in the brain of dogs naturally infected with *L*. *infantum* is parasite dependent or independent [22]. In this respect, the detection of viable forms of this parasite and its frequency in the CNS can help explain the cause of CNS inflammation observed in dogs with visceral leishmaniasis. In addition, the correlation of the presence and load of *L*. *infantum* with associated histological alterations in the CNS would permit a better understanding of the pathogenesis of this parasite in the CNS. Therefore, the aims of this study were to evaluate the occurrence, viability and load of *L*. *infantum* in the brain, spinal cord and CSF, and to identify the neurological histological alterations associated with this protozoan and its co-infections in naturally infected dogs from a ZVL-endemic area in Rio de Janeiro State, Brazil.

## Materials and methods

### Dog population

A descriptive study was conducted using a non-probabilistic sample of 48 dogs (28 males and 20 females) that were included from February to November 2014. Thirty-seven of these dogs were mongrels, three were Pinschers, two were Labrador Retrievers, two were German Spitz, one was a Shar Pei, one was a Dachshund, one was a Pit Bull, and one was a Poodle. The age of the dogs ranged from 1 to 7 years in 39 (81%) animals, five (11%) were older than 7 years, and four (8%) were less than 11 months old. The dogs were from the town of Barra Mansa (22°32’25.19” S and 44°10’35.33” W), Rio de Janeiro State, Brazil, a ZVL-endemic area with reports of cases in dogs [24] and humans [25]. All animals were privately owned and tested seropositive for anti-*Leishmania* antibodies by a rapid immunochromatographic test (Dual Path Platform, DPP^®^) and by enzyme immunoassay (ELISA). Both serological tests are produced by Bio-Manguinhos, Rio de Janeiro, Brazil. These tests were performed by public health services participating in the ZVL surveillance and control program of the state of Rio de Janeiro, with permission of the owners. In addition, tissue and CSF samples collected during necropsy of all dogs for parasitological confirmation were submitted to parasitological culture. Only dogs with isolation of *Leishmania* in parasitological culture, followed by identification of the species *L*. *infantum*, were included in the study. The species *L*. *infantum* was identified by multilocus enzyme electrophoresis (MLEE) in 23 dogs by testing one isolate from the following samples: skin, spleen, bone marrow, or lymph node. In addition, *L*. *infantum* was identified in all 48 dogs by quantitative real-time PCR (qPCR) in at least one of the following samples: spleen, brain, spinal cord, or CSF. None of the dogs studied had a history of chronic corticosteroid or anti-*Leishmania* drug use.

### Sample collection

Since they tested seropositive, the dogs were sent by the Municipal Health Department of Barra Mansa to be euthanized at Instituto Nacional de Infectologia Evandro Chagas (INI), Fundação Oswaldo Cruz, Brazil. The euthanasia procedure was performed according to the recommendations of the Brazilian Ministry of Health for the control of ZVL [3], with the owners providing signed consent. The dogs were not housed for any period of time prior to euthanasia. Immediately after arrival at INI, the dogs were submitted to physical examination that consisted of the inspection of behavior, alertness, posture, gait, skin and oral and ocular mucosae, as well as palpation of the superficial lymph nodes and organs. The animals were sedated by intramuscular administration of ketamine hydrochloride (10 mg/kg) and acepromazine maleate (0.2 mg/kg) and 1 to 3 mL blood was collected from the cephalic vein into sterile vacuum tubes without anticoagulant. After clotting, the blood samples were centrifuged at 1,125 × *g* and serum was separated and stored at -20°C until the time of analysis for indirect fluorescent antibody testing (IFAT) to detect anti-*Toxoplasma gondii* antibodies and for immunochromatographic testing to detect anti-*Ehrlichia canis* antibodies. Thereafter, the dogs were euthanized with an intravenous overdose of thiopental sodium, in compliance with the guidelines of the Federal Council on Veterinary Medicine of Brazil, and immediately necropsied.

During necropsy, 1 to 3 mL CSF was collected from the atlanto-occipital space using sterile needles and syringes. Part of this fluid was immediately seeded onto biphasic Novy, MacNeal and Nicolle (NNN)/Schneider’s Insect Medium (Sigma-Aldrich Co., St. Louis, MO, USA) containing 10% fetal bovine serum for the isolation of *Leishmania* in parasitological culture. The other part was transferred to sterile DNA-free polypropylene tubes and frozen at -20°C for qPCR, DPP^®^, and investigation of distemper virus antigens.

The CNS tissues were examined macroscopically. For parasitological culture, a pool of brain tissue samples (cerebrum, cerebellum, and brainstem), a pool of spinal cord tissue samples (cervical, thoracic, and lumbar regions), and spleen, skin, bone marrow and popliteal lymph node samples were collected aseptically and immersed in sterile saline. For qPCR in CNS tissues, a pool of brain tissue samples (cerebrum, cerebellum, and brainstem), a pool of spinal cord tissue samples (cervical, thoracic, and lumbar regions), and spleen samples were collected into sterile DNA-free polypropylene tubes and frozen at -20°C until the time of analysis. Additionally, fragments of the brain, cerebellum, brainstem, spinal cord (cervical, thoracic, and lumbar regions) and spleen were fixed in 10% neutral formalin and processed routinely for embedding in paraffin [26] for immunohistochemistry, *in situ* hybridization (ISH) and histopathology. The paraffin blocks of the spleen were only processed for immunohistochemistry. Only CNS tissues of dogs whose CNS samples had a positive culture result were submitted to ISH. Immunohistochemistry, ISH and histopathology were used to detect amastigote forms of *L*. *infantum* and to evaluate histological alterations associated with infection by this parasite.

The animals were tested for anti-*T*. *gondii* and anti-*E*. *canis* antibodies in serum and for distemper virus antigens in CSF because these pathogens can also infect and cause lesions in the CNS of dogs [27, 28].

### Parasitological culture for isolation and identification of the *Leishmania* species

The samples were seeded onto biphasic NNN/Schneider’s Insect Medium (Sigma-Aldrich Co., St. Louis, MO, USA) containing 10% fetal bovine serum and incubated at 26–28°C [29]. The *Leishmania* promastigotes isolated from skin, spleen, bone marrow and lymph nodes were identified as *L*. *infantum* by MLEE using five enzymatic systems [30]. These tests were performed at the Laboratório de Vigilância em Leishmanioses, INI, Fiocruz, which is a referral center for leishmaniasis of the Brazilian Ministry of Health.

### Quantitative real-time PCR for the diagnosis of *Leishmania infantum* and quantification of parasite load

Forty-eight brain samples, 48 spinal cord samples, 36 CSF samples, and 44 spleen samples were submitted to singleplex qPCR. DNA was extracted from the samples using the DNeasy^®^ Blood & Tissue Kit (Qiagen, Hilden, Germany) according to manufacturer recommendations. Tissue fragments ≤ 25 mg and 100 μL CSF were used. DNA was quantified in a Qubit^®^ 2.0 Fluorometer (Invitrogen, CA, USA) using the Qubit^®^ dsDNA HS Assay Kit (Thermo Fisher Scientific, Inc., MA, USA) according to manufacturer instructions. Amplification was performed with the StepOne™ System (Applied Biosystems, CA, USA) using primers LEISH-1 (5’-AACTTTTCTGGTCCTCCGGGTAG-3’) and LEISH-2 (5’-ACCCCCAGTTTCCCGCC-3’) and the TaqMan^®^ MGB probe (FAM- 5’AAAAATGGGTGCAGAAAT-3’-NFQ). The TaqMan-MGB probe and PCR primers were designed to target conserved regions of the *L*. *infantum* minicircle kinetoplast DNA. The cycling conditions were those described previously [31] using a final reaction volume of 25 μL. Each sample was processed in triplicate.

For the quantification of parasite load, a standard curve was constructed with serial dilutions (10^1^ to 10^5^ parasites) of *L*. *infantum* DNA (MHOM/BR/1974/PP75). Positive and negative controls were included in each amplification plate and a threshold of 0.1 was established. Samples in which DNA amplification occurred after the 37^th^ cycle were classified as undetectable. The *L*. *infantum* parasite load is expressed as the natural logarithm of the number of parasite genome equivalents (gEq)/ng.

Samples with undetectable results in the amplification were submitted to DNA quality testing using the TaqMan^®^ Gene Expression Assay (Applied Biosystems, CA, USA) in another singleplex qPCR. This technique consisted of a predefined pair of primers and a predefined probe for amplification of a segment of the canine gene encoding the β-actin subunit (Cf03023880_g1). A final reaction volume of 25 μL was used. The results are expressed as positive or negative and samples showing amplification were considered to be free of DNA degradation and PCR inhibitors.

### Detection of anti-*Leishmania* antibodies in cerebrospinal fluid by DPP^®^

Cerebrospinal fluid samples from 36 dogs were tested for the presence of anti-*L*. *infantum* antibodies by the DPP^®^ assay according to manufacturer recommendations for testing serum samples.

### Histopathology and immunohistochemistry

Serial sections (5 μm) were cut from the paraffin blocks containing the tissues and mounted on non-silanized slides for histopathology and on silanized slides for immunohistochemistry.

For histopathology, the tissues were stained with hematoxylin-eosin (HE) [26]. The inflammatory infiltrate in CNS tissues was classified as follows: granulomatous, predominance of macrophages; non-granulomatous, predominance of other types of inflammatory cells; suppurative, presence of neutrophils; non-suppurative, exclusive presence of mononuclear cells (lymphocytes, plasma cells, and macrophages).

For evaluation of inflammatory intensity, the cell types (macrophages, plasma cells, lymphocytes, eosinophils, and neutrophils) detected in the inflammatory infiltrate were analyzed semiquantitatively under a light microscope. For this purpose, the number of cells was determined at ×400 magnification in the most cellular area of the histological section using a 1-mm^2^ optical grid and a manual cell counter. The median inflammatory intensity was calculated for the entire inflammatory infiltrate (sum of all inflammatory cell types found) of each organ.

For immunohistochemistry, the slides were submitted to deparaffinization, rehydration, blocking of endogenous peroxidase, antigen retrieval, blockade of nonspecific protein binding, and incubation with polyclonal rabbit anti-*Leishmania* serum diluted 1:500 following a previously described protocol [32]. A polymer-based detection system (HiDef Detection HRP^TM^ Polymer System, Cell Marque, Rocklin, CA, USA) was used for the detection of amastigote forms of *Leishmania* spp. according to manufacturer recommendations.

### *In situ* hybridization

ISH was used to identify amastigote forms of *L*. *infantum* in the tissue sections by detecting a specific nucleic acid sequence of this parasite. For this purpose, an *L*. *infantum*-specific oligonucleotide probe labeled with digoxigenin that targets the minicircle kinetoplast DNA (kDNA) gene of the parasite [33] was used. Five-micrometer-thick sections were cut from the paraffin blocks and mounted on silanized slides. These sections were processed as described previously [32] using the ZytoFastPlus CISH Implementation Kit AP-NBT/BCIP^®^ (Zytovision GmbH, Bremerhaven, Germany). The probe was diluted 1:500 in Hybridization Solution H7782 (Sigma-Aldrich Co., St. Louis, MO, USA).

### Detection of canine distemper virus antigens in cerebrospinal fluid

Cerebrospinal fluid samples from 15 dogs with histological alterations in CNS tissues were investigated for distemper virus antigens using the rapid Alere Cinomose Ag Test Kit (Alere^TM^, São Paulo, Brazil), following the recommendations of the manufacturer for testing serum samples. According to the manufacturer, this test has a sensitivity of 98.8% and specificity of 97.7%.

### Detection of anti-*T*. *gondii* antibodies in serum

Serum samples from 14 out of 15 dogs with histological alterations in CNS tissues were tested for anti-*T*. *gondii* antibodies by IFAT according to a previously described protocol [34]. The serum sample of one of the dogs with histological alterations in CNS tissues was insufficient for the test. The conjugate used was Anti-Dog IgG (whole molecule)-FITC antibody produced in rabbits (Sigma-Aldrich Co., St. Louis, MO, USA). The sera were first diluted 1:16 and then at a ratio of four until 1:256. Samples with a titer of 1:16 or higher were classified as positive.

### Detection of anti-*Ehrlichia canis* antibodies in serum

Serum samples from 14 out of 15 dogs with histological alterations in CNS tissues were tested for anti-*E*. *canis* antibodies using the immunochromatographic Alere Erliquiose Ac Test Kit (Alere^TM^, São Paulo, Brazil), according to manufacturer instructions. The serum sample of one of the dogs with histological alterations in CNS tissues was insufficient for the test. According to the manufacturer, this test has a sensitivity of 98.2% and specificity of 100.0% and detects antibodies of the IgM and IgG classes.

### Statistical analysis

The data were analyzed using the R Project for Statistical Computing for Windows software (version 3.3). Simple frequencies of the following variables are reported: clinical classification (with or without clinical signs compatible with ZVL), clinical signs compatible with ZVL, dogs positive for *Leishmania* in CNS (according to the organ analyzed) and spleen, positive result in the diagnostic tests, classification of the inflammatory infiltrate, and histological alterations in the CNS and their locations.

The mean amplification efficiency, slope, y-intercept and R^2^ of qPCR were calculated using a threshold cycle (CT) value of at least 37 for positive results. Additionally, the median inflammatory intensity (sum of all inflammatory cell types found) of each organ and the median parasite load (expressed as the natural logarithm of the number of parasite genome equivalents (gEq)/ng) of all dogs studied were calculated. Spearman’s correlation coefficient was used to evaluate the correlation of parasite load in CNS tissues and spleen, with a coefficient higher than 0.4 indicating a good correlation. The nonparametric Kruskal-Wallis test and Mann-Whitney test with Bonferroni correction were applied to evaluate differences in parasite load of the CSF, brain, spinal cord and spleen. Additionally, the Mann-Whitney test was used to evaluate differences in parasite load of the brain and spinal cord between animals with and without histological alterations in the CNS and between dogs seropositive for *E*. *canis* and/or *T*. *gondii* and dogs seronegative for these two parasites, as well as differences in parasite load of the CSF, brain, spinal cord and spleen between animals testing positive and negative by the parasitological techniques (culture and histological techniques) in the CNS. A p value < 0.05 was considered significant in the tests. Boxplots and strip plots were used to aid with the comparison of parasite load in the different samples studied. Additionally, the sensitivity, specificity and accuracy of the DPP^®^ assay were calculated using the results of parasitological culture and qPCR in CSF as a composite reference standard.

### Ethics statement

This study was carried out in strict accordance with the recommendations of the Brazilian Ministry of Health and of the Federal Council on Veterinary Medicine of Brazil, with permission of the owners. The study protocol was approved by the Ethics Committee on Animal Use of the Oswaldo Cruz Foundation (CEUA/FIOCRUZ; Permit Number: LW-54/13). All blood samples were collected under sedation with ketamine hydrochloride and acepromazine maleate. The dogs were euthanized with an intravenous overdose of thiopental sodium and all efforts were made to minimize suffering.

## Results

Forty (83%) of the 48 dogs analyzed exhibited clinical signs compatible with ZVL. The following clinical signs compatible with ZVL were observed: furfuraceous desquamation of skin (44%), onychogryphosis (44%), weight loss (42%), opaque hair (42%), dehydration (33%), regional lymphadenomegaly (33%), local alopecia (29%), pale mucosae (29%), splenomegaly (29%), ophthalmic alterations (27%), apathy (27%), cachexia (13%), generalized alopecia (13%), generalized lymphadenomegaly (10%), skin ulcers (8%), hepatomegaly (8%), limb edema (8%), epistaxis (4%), arthralgia (2%), jaundice (2%), and pain when palpating the kidneys (2%). Neurological clinical signs were not observed.

**Fig 1:**
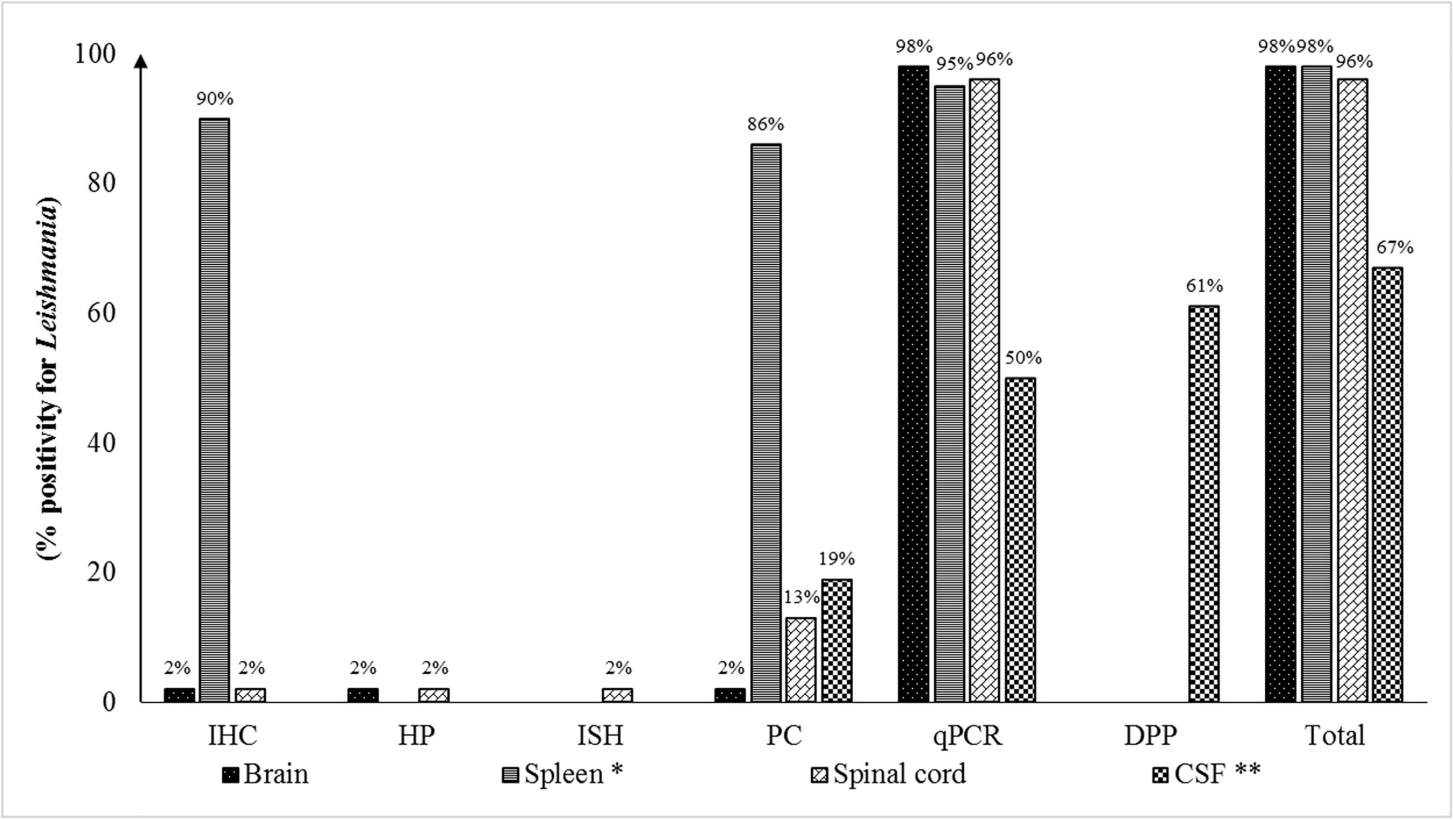
Frequency of *Leishmania* positivity in the central nervous system and spleen of naturally infected dogs detected by histopathology (HP), immunohistochemistry (IHC), *in situ* hybridization (ISH), parasitological culture (PC), DPP^®^ assay, and qualitative real-time PCR (qPCR). * 44 samples were investigated by qPCR; ** 36 samples were investigated by qPCR and DPP^®^ assay. CSF = cerebrospinal fluid.

All 48 dogs examined tested positive for *L*. *infantum* in the CNS by at least one of the diagnostic techniques used (Fig 1).

Using parasitological culture, 12 dogs (25%) tested positive in at least one CNS sample. Six (50%) of these 12 dogs were positive only in CSF, three (25%) in CSF and spinal cord, two (17%) only in the spinal cord, and one (8%) in spinal cord and brain.

Quantitative real-time PCR detected *L*. *infantum* DNA in at least one CNS sample in all animals (Fig 1). All samples exhibited a positive result in the DNA quality test, indicating the absence of DNA degradation and PCR inhibitors. One brain sample, two spinal cord samples, and 18 CSF samples tested negative by this technique. Among the 18 CSF samples that were negative by qPCR, nine (50%) were positive for *Leishmania* only in the DPP^**®**^ assay and one (6%) was positive only in parasitological culture. All brain and spinal cord samples that were negative by qPCR tested also negative for *Leishmania* by the other techniques.

**Fig 2:**
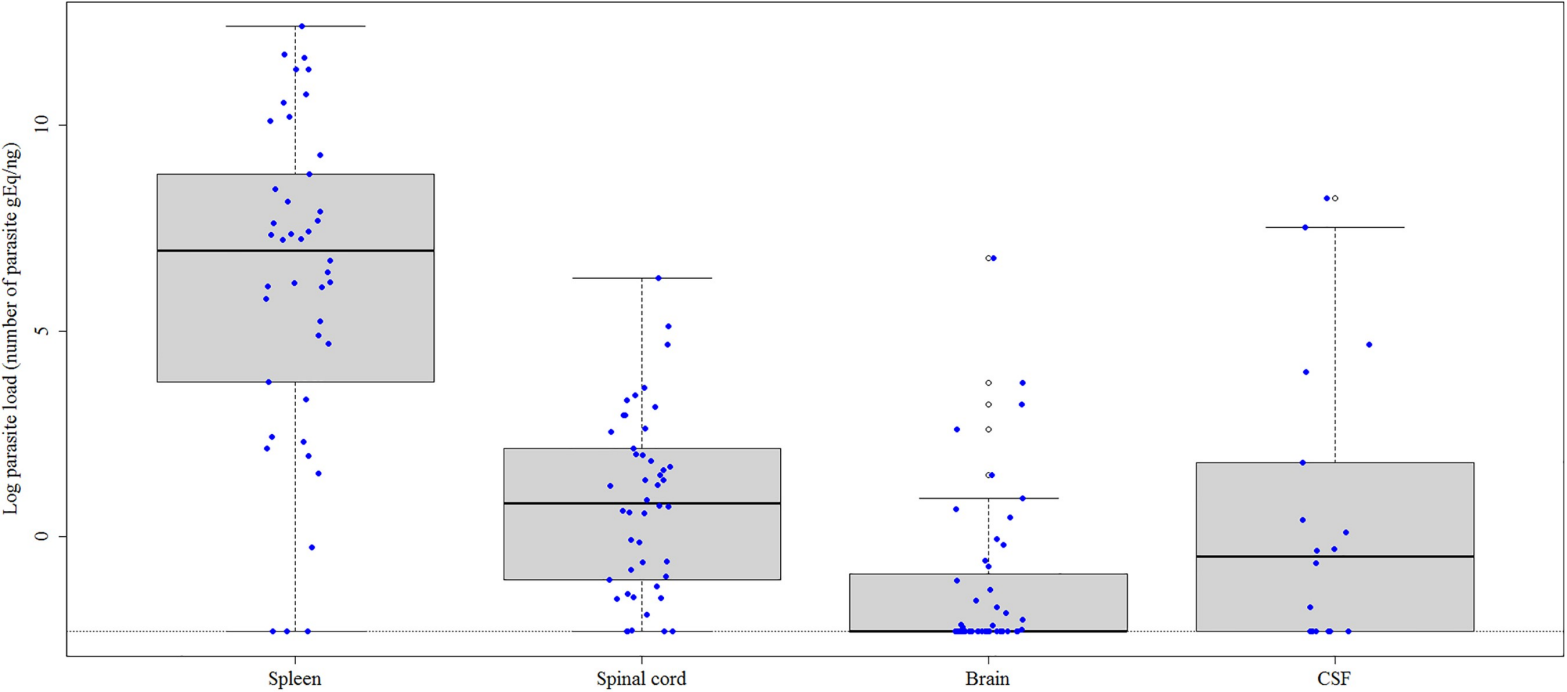
*Leishmania infantum* parasite load expressed as the natural logarithm of the number of parasite genome equivalents (gEq)/ng in the brain, spinal cord, cerebrospinal fluid and spleen of naturally infected dogs. The horizontal black lines indicate the median parasite load. The vertical dotted lines indicate the interquartile range. The horizontal dotted lines indicate the lower limit of positivity (threshold). The blue dots represent the parasite load of each dog. The empty dots are the outliers of parasite load defined by the boxplot. CSF = cerebrospinal fluid.

The median *L*. *infantum* parasite load was 6.961 in the spleen, 0.823 in the spinal cord, -0.486 in the CSF, and -2.302 in the brain (Fig 2). The parasite load was significantly higher in the spleen than in spinal cord, brain and CSF (p < 0.001) and was higher in the spinal cord than in the brain (p < 0.001). There was no significant correlation between parasite load in the spleen and CNS samples.

**Fig 3:**
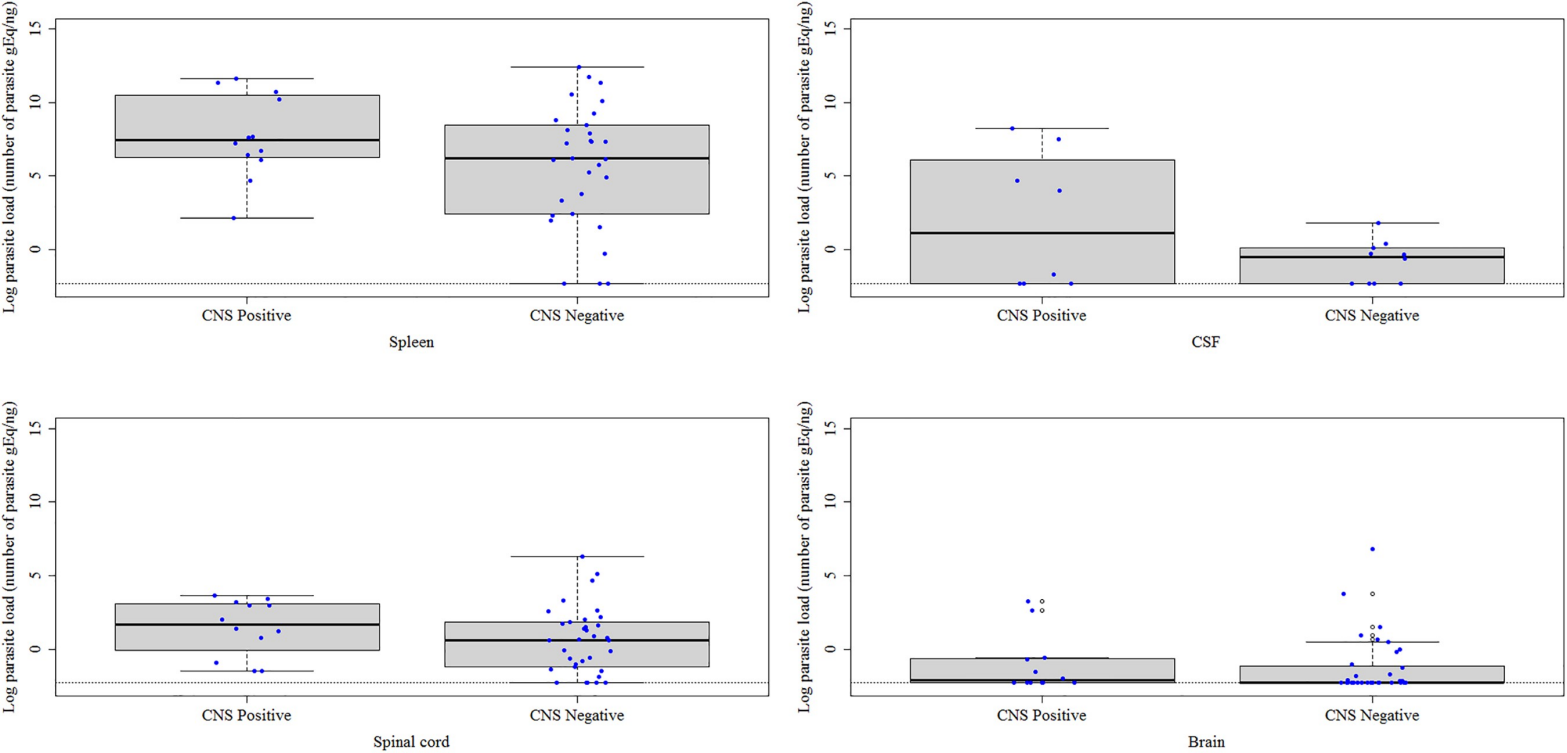
*Leishmania infantum* parasite load expressed as the natural logarithm of the number of parasite genome equivalents (gEq)/ng in the central nervous system (CNS) and spleen of naturally infected dogs according to a positive or negative *L*. *infantum* result in the CNS by the parasitological techniques (culture and histological techniques). The horizontal black lines indicate the median parasite load. The vertical dotted lines indicate the interquartile range. The horizontal dotted lines indicate the lower limit of positivity (threshold). The blue dots represent the parasite load of each dog. The empty dots are the outliers of parasite load defined by the boxplot. CSF = cerebrospinal fluid. CNS = central nervous system.

Fig 3 shows the parasite load in the CNS and spleen of dogs according to a positive or negative result for *L*. *infantum* in the CNS by the parasitological techniques. The median parasite load of dogs positive for *L*. *infantum* was 7.427 in the spleen, 1.685 in the spinal cord, 1.151 in the CSF, and -2.133 in the brain. In *L*. *infantum*-negative dogs, the median parasite load was 6.185 in the spleen, 0.616 in the spinal cord, -0.484 in the CSF, and -2.303 in the brain. No significant differences were observed between these groups.

The sensitivity, specificity and accuracy of the DPP^®^ assay in detecting anti-*Leishmania* antibodies in CSF samples were 68% (56–81%), 47% (34–60%) and 58% (45–71%), respectively, considering a 95% confidence interval.

**Fig 4:**
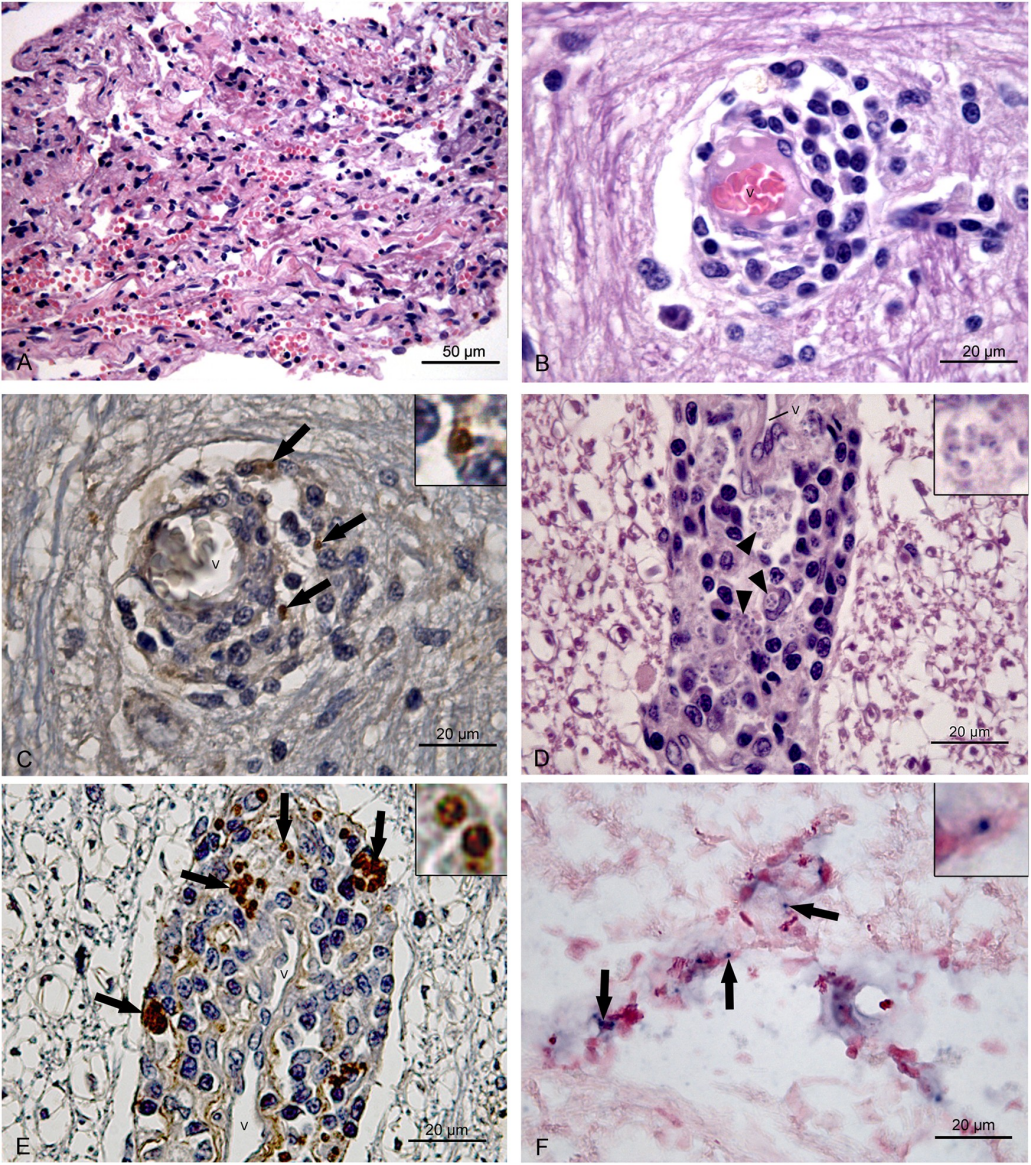
Histological findings in the central nervous system of dogs naturally infected with *Leishmania infantum*. V = vascular lumen. (A-C) Brainstem. (A) Suppurative meningitis consisting of lymphocytes, macrophages, and neutrophils. HE. (B) Perivascular cuffing consisting of lymphocytes, plasma cells, and macrophages in the grey matter. HE. (C) Brown-stained amastigote forms of *Leishmania* (arrows) in the perivascular space of grey matter. IHC. (D-F) White matter of the cervical spinal cord. (D) Several amastigote forms of *Leishmania* in the cytoplasm of macrophages (arrowhead) and a granulomatous inflammatory infiltrate consisting of lymphocytes, macrophages and plasma cell in the perivascular space. HE. (E) Brown-stained amastigote forms of *Leishmania* (arrows) in the perivascular space. IHC. (F) Blue-stained amastigote forms of *Leishmania infantum* (arrows) in the perivascular space. ISH.

Gross examination of CNS tissues revealed no alterations. Histological analysis detected inflammatory alterations in CNS tissues of 15 (31%) dogs. In these 15 dogs, meningitis was observed in 12 (80%) animals (Fig 4A), choroid plexitis in four (27%), encephalitis in one (7%), and myelitis in one (7%). Encephalitis and myelitis were characterized by perivascular cuffing (Fig 4B and 4D). A granulomatous non-suppurative infiltrate was observed in 13 (87%) dogs, a non-granulomatous non-suppurative (lymphoplasmacytic) infiltrate in four (27%), and a granulomatous suppurative infiltrate in three (20%).

In one of the 15 dogs with inflammatory alterations in the CNS, amastigote forms of *Leishmania* spp. were observed in the cytoplasm of macrophages in the perivascular space of the brainstem grey matter (Fig 4C) and in cervical spinal cord white matter (lateral and ventral funiculi) (Fig 4D–4F) and grey matter. The inflammatory infiltrate associated with the amastigote forms in the perivascular space was of the granulomatous non-suppurative type (Fig 4B and 4D). Additionally, *L*. *infantum* was detected in the spinal cord and CSF of this dog by parasitological culture and in the spleen by immunohistochemistry and parasitological culture. The parasite load was 11.364 in the spleen, 4.007 in the CSF, 3.228 in the brain and -1.471 in the spinal cord. This dog was a 3-year-old male German Spitz that had no neurological sign, but exhibited pale mucosae, local alopecia and onychogryphosis.

**Fig 5:**
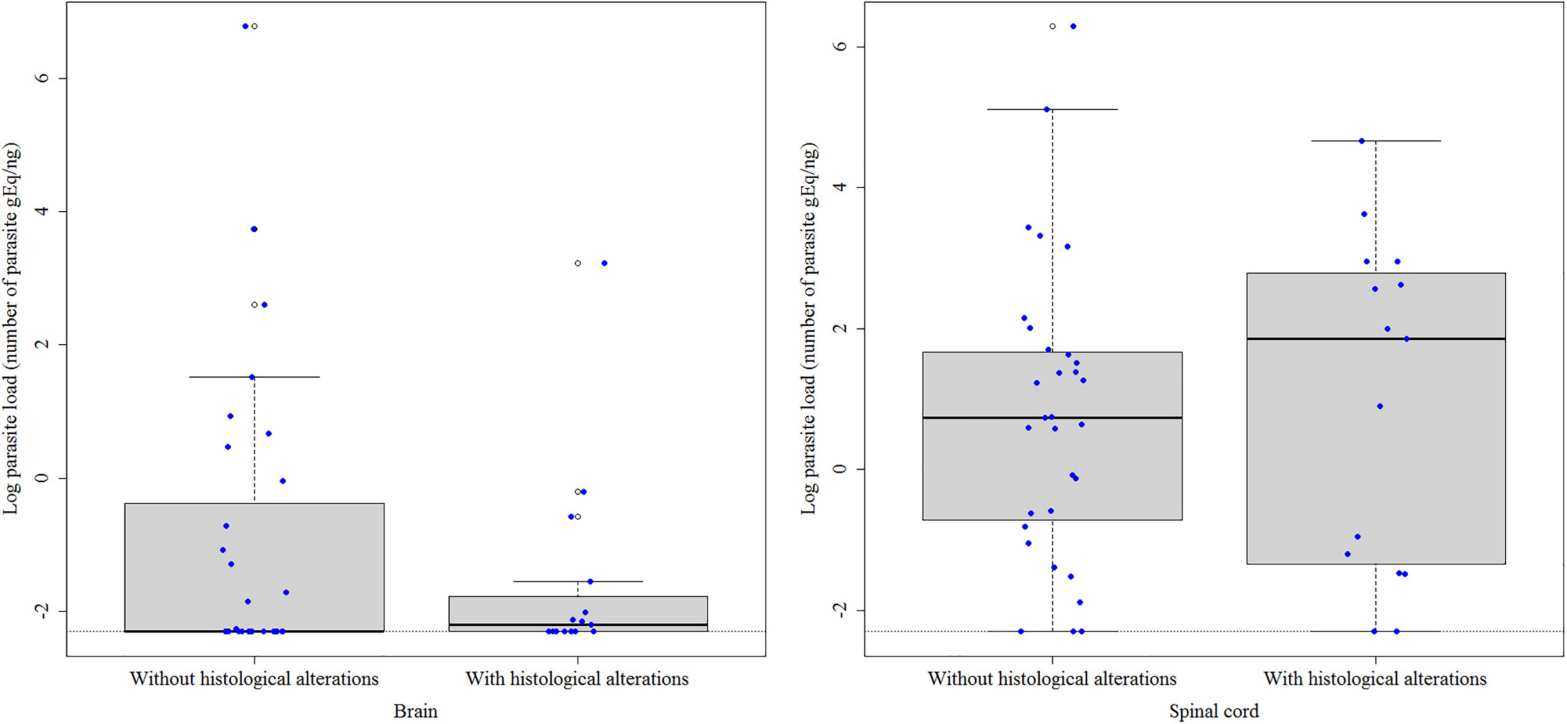
*Leishmania infantum* parasite load expressed as the natural logarithm of the number of parasite genome equivalents (gEq)/ng in the brain and spinal cord of naturally infected dogs with and without histological alterations in the central nervous system. The horizontal black lines indicate the median parasite load. The vertical dotted lines indicate the interquartile range. The horizontal dotted lines indicate the lower limit of positivity (threshold). The blue dots represent the parasite load of each dog. The empty dots are the outliers of parasite load defined by the boxplot.

The animals were divided into groups with (15 dogs) and without histological alterations (33 dogs) in the CNS. The median parasite load of dogs with histological alterations was 1.856 in the spinal cord and -2.192 in the brain (Fig 5). In dogs without histological alterations, the median parasite load was 0.738 in the spinal cord and -2.303 in the brain (Fig 5). No significant differences were observed between these groups.

None of the 15 dogs with inflammatory alterations in the CNS exhibited histological alterations compatible with distemper virus infection or was positive for antigens of this virus in CSF. Fourteen of these 15 dogs were submitted to the investigation of anti-*T*. *gondii* and anti*-E*. *canis* antibodies in serum. Four (28.6%) dogs were positive for anti-*T*. *gondii* and anti-*E*. *canis* antibodies, four (28.6%) were positive for anti-*T*. *gondii* antibodies, four (28.6%) were negative for both antibodies, and two (14.2%) were positive for anti-*E*. *canis* antibodies. The anti-*T*. *gondii* antibody titers ranged from 1:16 to 1:64. *T*. *gondii* tachyzoites or bradyzoite-containing tissue cysts and *Babesia* spp. parasitizing erythrocytes in capillary beds were not detected in CNS tissues by histopathology in any of the dogs. One of the four dogs with negative anti-*T*. *gondii* and anti-*E*. *canis* antibodies was the German Spitz dog exhibiting amastigote forms associated with granulomatous non-suppurative encephalomyelitis. Among the 14 dogs, viable forms of *L*. *infantum* were isolated by parasitological culture in three dogs positive for both anti-*T*. *gondii* and anti-*E*. *canis* antibodies and in two dogs with a negative anti-*T*. *gondii* and anti-*E*. *canis* antibody result.

Table 1 shows the intensity of inflammatory cells/mm^2^ in different CNS tissues of 14 dogs infected with *L*. *infantum* and with inflammatory alterations in the CNS according to the results of serum anti-*T*. *gondii* and anti-*E*. *canis* antibody testing.

**Table 1:** Intensity of inflammatory cells/mm^2^ in different CNS tissues of 14 dogs with inflammatory alterations in the CNS and naturally infected with *L*. *infantum* according to serum positivity for anti-*E*. *canis* and anti-*T*. *gondii* antibodies.

Tissue sample	Intensity of inflammatory cells/mm^2^							
	*L*. *infantum* positive^a^ (N = 4)		*L*. *infantum + E*. *canis* positive (N = 2)		*L*. *infantum + T*. *gondii* positive (N = 4)		*L*. *infantum + T*. *gondii + E*. *canis* positive (N = 4)	
	Median	Range	Median	Range	Median	Range	Median	Range
**Cerebrum**	0	0–39	0.5	0–50	0	0–20	0	0–173
**Cerebellum**	0	0–101	17	0–94	0	0–0	20.5	0–53
**Brainstem**	0	0–34	0	0–0	0	0–53	4.5	0–224
**Cervical S.C.**	0	0–67	0	0–28	0	0–0	3	0–38
**Thoracic S.C.**	0	0–0	0	0–15	0	0–0	0	0–102
**Lumbar S.C.**	0	0–0	0	0–12	0	0–86	0	0–62

S.C., spinal cord; N, number of dogs.

^a^ Dogs positive only for *L*. *infantum*.

**Fig 6:**
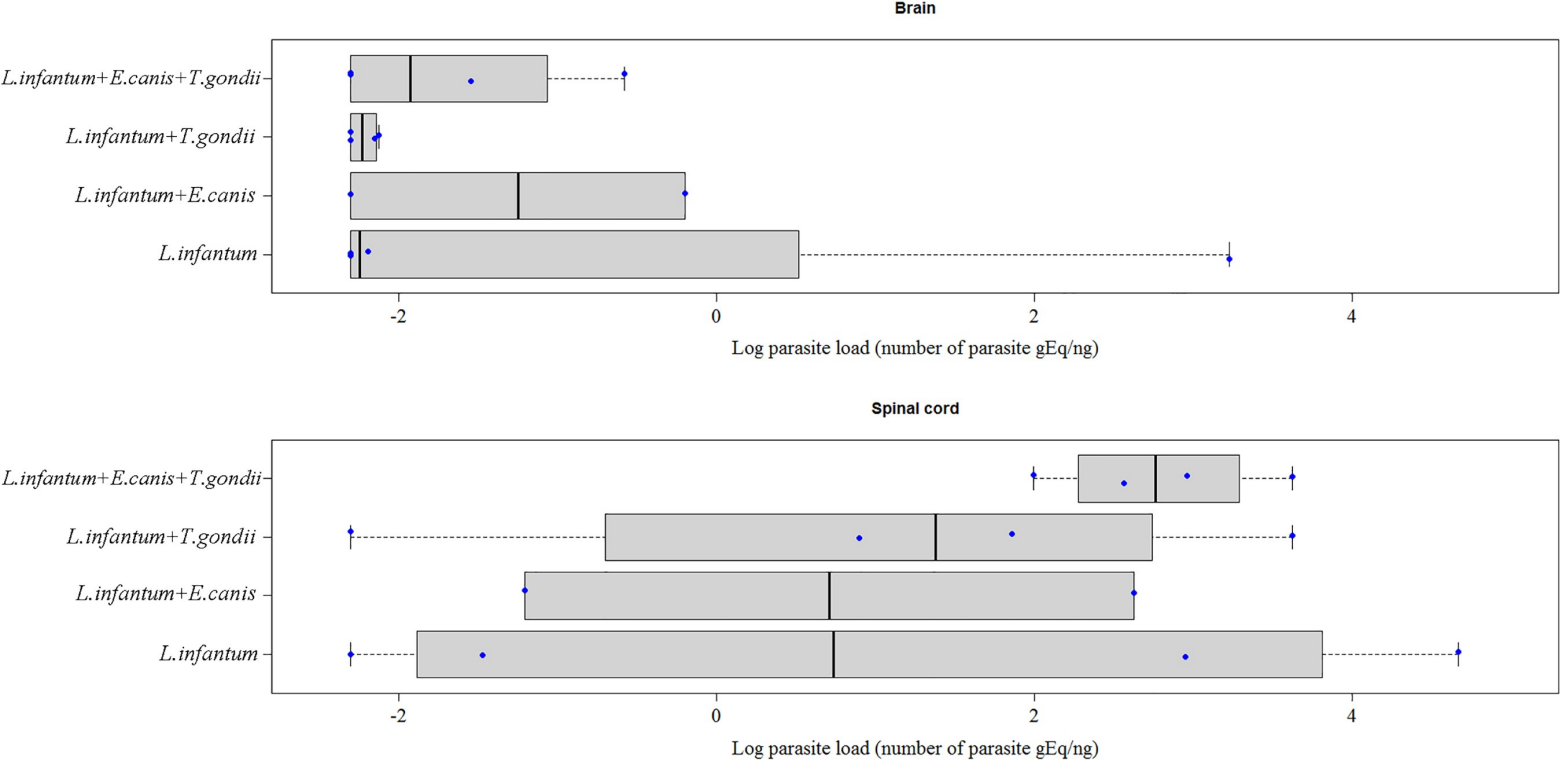
*Leishmania infantum* parasite load expressed as the natural logarithm of the number of parasite genome equivalents (gEq)/ng in the brain and spinal cord of 14 dogs with inflammatory alterations in the CNS according to serum positivity for anti-*E*. *canis* and anti-*T*. *gondii* antibodies. The vertical black lines indicate the median parasite load. The horizontal dotted lines indicate the interquartile range. The blue dots represent the parasite load of each dog.

For the 14 dogs with inflammatory alterations in CNS, Fig 6 shows the median parasite load in CNS tissues according to serum positivity for anti-*E*. *canis* and anti-*T*. *gondii* antibodies. In the four dogs positive only for *L*. *infantum*, the median parasite load was -2.247 in the brain and 0.738 in the spinal cord. In the four dogs positive for *L*. *infantum*, *E*. *canis* and *T*. *gondii*, the median parasite load was **-**1.925 in the brain and 2.760 in the spinal cord. In the four dogs positive only for *L*. *infantum* and *T*. *gondii*, the median parasite load was -2.228 in the brain and 1.377 in the spinal cord. In the two dogs positive only for *L*. *infantum* and *E*. *canis*, the median parasite load was -1.250 in the brain and 0.711 in the spinal cord. There were no significant differences in the median parasite load between dogs seropositive for *E*. *canis* and/or *T*. *gondii* and dogs seronegative for these two parasites.

## Discussion

The frequency of *L*. *infantum* DNA was very high in the brain and spinal cord of the dogs evaluated. The present results and those of other studies in the brain [13, 20, 22] suggest the occurrence of *L*. *infantum* DNA to be common in the CNS of dogs naturally infected with this parasite.

Despite the frequent occurrence of *L*. *infantum* DNA in the CNS of infected dogs, amastigote forms of this parasite were rarely detected in the present study. The detection of amastigote forms of *L*. *infantum* in the CNS of dogs had only been described in Europe in rare reports of cases in the meninges [16], choroid plexus [11, 15], thalamus [11], spinal nerves [11], spinal canal granuloma [35], and spinal cord parenchyma [11].

In addition to the detection of amastigote forms, the occurrence of active infection in the CNS was also confirmed by parasitological culture that detected viable forms of this parasite mainly in CSF samples. These results indicate that *L*. *infantum* can spread through CSF to the entire CNS of dogs. The sensitivity of parasitological culture of CSF would have been even higher if an enrichment method consisting of gentle centrifugation (500–800 rpm) and collection and seeding of pellets in culture medium were used. Immunosuppression triggered by the long-term use of corticosteroids in a tetraplegic dog [11] and parasite escape from the action of an anti-*Leishmania* drug in a child with neurological signs [36] have been indicated as possible causes for the invasion of viable forms of *Leishmania* into the CNS. In contrast to these hypotheses, in the present study in which the dogs were not treated with anti-*Leishmania* drugs or corticosteroids, 25% of the animals exhibited viable forms of *L*. *infantum* in the CNS.

The frequency of anti-*Leishmania* antibodies in CSF of the dogs studied is close to the frequencies reported by other authors of 69% [5] and 62% [20] using ELISA in naturally infected dogs. However, the sensitivity and specificity of the DPP^®^ assay were lower than those observed for serum samples of naturally infected dogs (83% and 73%, respectively) [37]. The detection of antibodies in CSF indicates disruption of the blood-brain barrier or blood-CSF barrier [38].

The inflammatory histological alterations observed here in the CNS are common findings reported in previous studies on dogs naturally infected with *L*. *infantum* [7, 9, 11, 12, 15–19, 39]. However, these alterations are not specific for infection with *L*. *infantum* and it is therefore not possible to demonstrate their association with parasitism. The detection of anti-*T*. *gondii* and anti-*E*. *canis* antibodies in the serum of more than half of the dogs with inflammatory alterations suggests that these parasites also contributed to these alterations since they can cause inflammatory infiltrates in the CNS similar to those observed in this study [27, 28]. The higher intensity of the inflammatory infiltrate in dogs seropositive for *E*. *canis* and *T*. *gondii* compared to dogs seronegative for these two pathogens supports this hypothesis. However, an association of granulomatous non-suppurative encephalomyelitis with *L*. *infantum* parasitism was demonstrated in one dog by the detection of amastigote forms in macrophages of the inflammatory infiltrate. In addition, there was no evidence of co-infection with other pathogens in the CNS of this dog. Granulomatous inflammatory infiltration of the parasitized CNS by amastigote forms of *Leishmania* has also been reported by other authors [11, 15, 16, 35, 40]. Although amastigote forms were not detected in the CNS of the other dogs of this study, the participation of *L*. *infantum* in the formation of the inflammatory alterations seen in the CNS cannot be ruled out. This suggestion is supported by the observation that the CNS of all dogs was infected with *L*. *infantum* and that the median parasite load of animals with histological alterations in the CNS was higher than that of animals without histological alterations, although the difference was not significant. Furthermore, even in the absence of amastigote forms, inflammatory lesions can be triggered when peripheral stimuli such as parasite antigens or DNA or inflammatory mediators reach the CNS and activate the inflammatory process [22].

The highest median parasite load in the CNS was identified in the spinal cord. Hemorrhage, granulomas, vasculitis, and necrosis have been reported in the spinal cord of dogs with canine ZVL [7, 11, 12, 35]. These histological alterations and the findings of this study regarding parasite load and occurrence of amastigote forms, associated with the histological alterations in this organ, suggest the spinal cord to be one of the areas of the CNS that are most susceptible to infection with this parasite.

Infection with *E*. *canis* can cause immunosuppression in dogs [41], a fact that could explain the higher *L*. *infantum* loads in the brain of dogs seropositive for this parasite when compared to animals that were seronegative for *T*. *gondii* and *E*. *canis*. On the other hand, *L*. *infantum* also causes immunosuppression [42] and the higher loads of this parasite observed in the CNS of dogs seropositive for *T*. *gondii* and/or *E*. *canis* may have rendered these animals more susceptible to infection and reactivation of infection with these two pathogens. However, co-infection was not associated with the occurrence of neurological signs in the present study or in the study of Cardinot et al. [13], nor with higher *L*. *infantum* viability in this study.

## Conclusions

The protozoan *L*. *infantum* can cross the blood-brain barrier, spread through CSF and cause active infection in the brain and spinal cord of dogs naturally infected with this parasite without a history of chronic corticosteroid or anti-*Leishmania* drug use. Additionally, this parasite can cause granulomatous non-suppurative encephalomyelitis, a condition that can lead to neurological clinical signs with progression of the disease. The spinal cord is an important site of infection with *L*. *infantum* in the CNS and should be investigated in cases of dogs with neurological clinical signs from ZVL-endemic areas.
