# Correction: Occurrence of *Leishmania infantum* in the central nervous system of naturally infected dogs: Parasite load, viability, co-infections and histological alterations

**DOI:** 10.1371/journal.pone.0188150

**Published:** 2017-11-14

**Authors:** The PLOS ONE Staff

The following information is missing from the Funding section: This study was supported by Coordenação de Aperfeiçoamento de Pessoal de Nível Superior (http://www.capes.gov.br/). The publisher apologizes for the error.
